# Clinical and radiological features that predict malignant transformation in cystic lesions of the pancreas: a retrospective case note review

**DOI:** 10.12688/amrcopenres.12860.2

**Published:** 2020-01-16

**Authors:** Margaret G. Keane, Hannah R. Dadds, Ghassan El Sayed, Tu Vinh Luong, Brian R. Davidson, Guiseppe K. Fusai, Douglas Thorburn, Stephen P. Pereira

**Affiliations:** 1 Institute for Liver and Digestive Health, University College London, London, NW3 2QG, UK; 2 Department of Gastroenterology, University College London Hospitals NHS Trust, London, UK; 3 Department of Cellular Pathology, University College London, London, UK; 4 Department of HPB Surgery, Royal Free Hospital, London, NW3 2QG, UK

**Keywords:** Pancreatic cystic lesion, IPMN, MCN, SCN, SPN, Pancreatic cancer, PNET

## Abstract

**Background:** Pancreatic cystic lesions (PCL) are being detected with increasing frequency. Current methods of stratifying risk of malignant transformation are imperfect. This study aimed to determine the frequency of pancreatic malignancy in patients with PCL and define clinical and radiological features that predict malignant transformation in patients managed by surgery and/or surveillance.

**Methods: **A retrospective cohort of adults who were evaluated in a tertiary hepatopancreaticobiliary centre between January 2000 - December 2013 with a confirmed PCL and followed up for at least 5 years. All cystic lesions were discussed at a weekly multidisciplinary meeting.

**Results: **Of the 1,090 patients diagnosed with a PCL, 768 patients were included in the study: 141 patients were referred for immediate pancreatic resection, 570 entered surveillance while 57 had a malignant PCL which was unresectable at diagnosis (n=47) or were unfit for surgery (n=10). In those who were resected following presentation, malignancy was present in 38%. During follow-up 2% of those entering a surveillance programme underwent malignant transformation. Clinical and radiological features associated with a high-risk PCL included older age, symptoms, associated solid component or dilated main pancreatic duct. In intraductal papillary mucinous neoplasms, larger size was not a feature of malignant transformation (benign vs. malignant 30mm vs. 23mm; P= 0.012).

**Conclusion: **The sensitivity of standard diagnostic tests leading to immediate surgery for high-risk PCL (malignant or mucinous) was 92% but with a specificity of just 5%. Surveillance of PCL without high-risk features within a multidisciplinary meeting was associated with a low incidence of cancer development, supporting the use of worrisome clinical and radiological features in the initial stratification of PCL.

## Introduction

Pancreatic cystic lesions (PCL) have become an increasingly common radiological finding, due largely to a greater availability and sensitivity of cross-sectional imaging. PCL are identified in 1.2–2.6%^1^,^2^ of patients undergoing abdominal computed tomography (CT) and in up to 13.5% of patients undergoing an MRI for non-pancreatic indications^3^. PCL can represent a range of different lesions; the most common lesions are classified by the World Health Organisation pathologically as an intraductal papillary mucinous neoplasm (IPMN), mucinous cystic neoplasm (MCN), serous cystadenoma (SCA), solid pseudopapillary neoplasm (SPN), cystic pancreatic neuroendocrine tumour (PNET) or cystic degeneration of pancreatic ductal adenoacarcinoma (PDAC)^4^.

It is estimated that 8% of all cases of PDAC arise from a PCL^5^. Detection therefore offers a significant opportunity for early curative intervention in a disease with a dismal prognosis. However the prevalence of these lesions is high and their natural history remains poorly understood. Malignant transformation of premalignant lesions is estimated to occur at a rate of approximately 0.95% per year^6^ and studies that have followed mucinous lesions long-term, have suggested that those that undergo malignant transformation takes at least 5 to 10 years to develop invasive disease^7^,^8^.

In accordance with international guidance, patients with PCL that are thought to be malignant or that are at high-risk of malignant transformation are referred for immediate surgical resection while other patients undergo regular surveillance with interval imaging^9^. In surveillance cohorts, PCL are usually smaller and without worrisome features. The exact clinical and radiological features, which predict mucinous subtypes that mandate, follow up or features which suggest malignant transformation, continue to be debated^9^–^14^. Recent retrospective cohort studies have shown that the 2012 international guidelines are more accurate than earlier guidance when triaging patients with PCL (PPV 88% vs. 67%, NPV 93% vs. 88%)^15^,^16^, but highlighted that low-grade malignant lesions such as SPN, PNET and occasionally benign mucinous tumours can still be misclassified as no-risk or low-risk lesions using these criteria. Further studies from large patient cohorts who have undergone careful classification and long-term follow up are therefore required.

### Study aims and objectives

The primary aim of this study was to predict the features of malignant transformation in a cohort of UK patients with a PCL referred to a tertiary hepatopancreaticobiliary (HPB) multidisciplinary team meeting (MDT).

## Methods

### Ethical consideration

The study protocol was reviewed by the Health Research Authority and deemed to primarily be an audit of current practice and therefore formal ethical review was not required.

### Setting

A large regional hepatopancreaticobiliary cancer centre based across two tertiary-care hospitals; University College Hospital and the Royal Free Hospital, London.

### Design

Retrospective case-note review.

### Management

In the UK there are no national guidelines for the management of PCL so management is performed in accordance with the published International, European and AGA guidelines^9^,^17^. As management varies between these guidelines our centre has elected to discuss all cases of PCL at a weekly MDT meeting.

If the PCL is associated with no worrisome features, surveillance with interval imaging is the favoured management strategy. However patients with larger PCL with features suspicious of malignant transformation (solid component, dilated main pancreatic duct >6mm or associated symptoms e.g. jaundice) and without significant comorbidity were referred for surgery. Patients with features of malignant transformation but who were unsuitable for surgical resection (due to comorbidity or extent of disease) typically had the diagnosis confirmed histologically and were referred for oncological or palliative care.

### Study definitions

A symptomatic PCL was defined as a lesion identified on imaging performed for the evaluation of attributable upper abdominal pain, obstructive jaundice or acute pancreatitis. For malignant lesions, weight loss, back pain and new-onset or deterioration of diabetes were also recognised to be associated symptoms.

If multiple PCL were present, the characteristics of the most significant cyst were reported (i.e. the largest cyst or the cyst with associated worrisome features).

Serum carcinoembryonic antigen (CEA) level >4.0 ng/mL and serum carbohydrate antigen 19-9 (CA 19-9) level >37 U/mL were considered to be elevated. In this study, all mixed type IPMNs (MT-IPMN) i.e. IPMN lesions which met criteria of both main duct and side branch lesions, were considered as main duct IPMNs (MD-IPMN). The PCL were further sub-classified on the basis of the most aggressive histological epithelial changes in accordance with the World Health Organisation (WHO) classification system^4^. Tumours were graded as having low or intermediate grade dysplasia, high-grade dysplasia including carcinoma *in situ* and malignant when invasive carcinoma was present, in line with the updated WHO classification of PCL^4^. Length of follow-up for the surveillance group was calculated from the time of the first cross-sectional imaging to the last cyst-related outpatient appointment. If patients did not attend clinic for any reason, interval imaging was used to define the last point of contact and to calculate the length of follow-up.

### Inclusion criteria

Patients diagnosed with a PCL between January 1^st^ 2000 and December 31^st^ 2013 were included. Cases were identified primarily from records of the weekly HPB multidisciplinary team (MDT) meetings. In addition, the Pathology (CoPath histology database, Sunquest, Tucson AZ, USA), Endoscopy (GI reporting tool, Unisoft medical systems, UK) and Imaging (PACS: picture archiving and communication system, GE Healthcare, USA) databases were searched using the following terms; pancreatic cyst, serous cystadenoma, intraductal papillary mucinous neoplasm, mucinous cystic neoplasm, mucinous cyst adenocarcinoma, solid pseudopapillary neoplasm, cystic pancreatic neuroendocrine tumour.

### Exclusion criteria

After initial review, the following patients were excluded from the final analysis: patients < 18 years, patients with solid lesions, patients without cross-sectional imaging confirming the presence of a pancreatic cyst, patients with a confirmed inflammatory pancreatic cyst - defined as a cyst measuring more than 4cm on CT/MRCP and located within or adjacent to the pancreas with a documented history of acute or chronic pancreatitis. Four of these patients had an inflammatory cyst proximal to an obstructing pancreatic tumour but none developed *de-novo* pancreatic cancer during a median follow-up of 12 months.

### Data recorded

Electronic medical records of the included patients were reviewed and information was recorded in an electronic spreadsheet; data obtained included demographic information (age, sex, hospital number), initial symptoms, history of pancreatitis or solid organ malignancy, family history of pancreatic cancer or relevant clinical syndrome. Recorded laboratory data including elevations in serum amylase, CEA and CA19-9. Baseline imaging (ultrasound, computed tomography (CT), magnetic resonance cholangiopancreatography (MRCP)), and endoscopic studies (endoscopic retrograde cholangiopancreatography (ERCP) and endoscopic ultrasound (EUS) with or without fine needle aspiration (FNA)) used in diagnosis were recorded. Features recorded from cross-sectional imaging included date of examination, size (maximal dimension), location and number of cystic lesions, presence of a solid component (mural nodules, solid component, calcification of the cyst or the wall, wall thickening), presence of septations, features of acute or chronic pancreatitis, dilatation of the pancreatic duct or biliary tree and communication of the cystic lesion with the main pancreatic duct or a side branch. For patients undergoing EUS-FNA or ERCP, imaging features at the time of the procedure were recorded as well as cytology, histology and biochemistry (CEA and amylase) results. Results of percutaneous biopsies or PET-CT scans were also recorded. For patients referred for surgery, date of operation, type of resection, final histology and length of follow-up including frequency of post-operative imaging, were recorded. For patients entered into a surveillance programme, length of surveillance, frequency of imaging and changes in size of the lesion during surveillance was also recorded.

### Statistical analyses

Statistical Package for Social Sciences for Windows, version 18.0 (SPSS Inc., Chicago, IL, USA) was used to perform all statistical analyses. Associations between malignancy and various clinical and radiographic characteristics were evaluated using a 2-sample *t* test for continuous variables, and a Chi-squared test for categorical variables.

## Results

**Figure 1.**
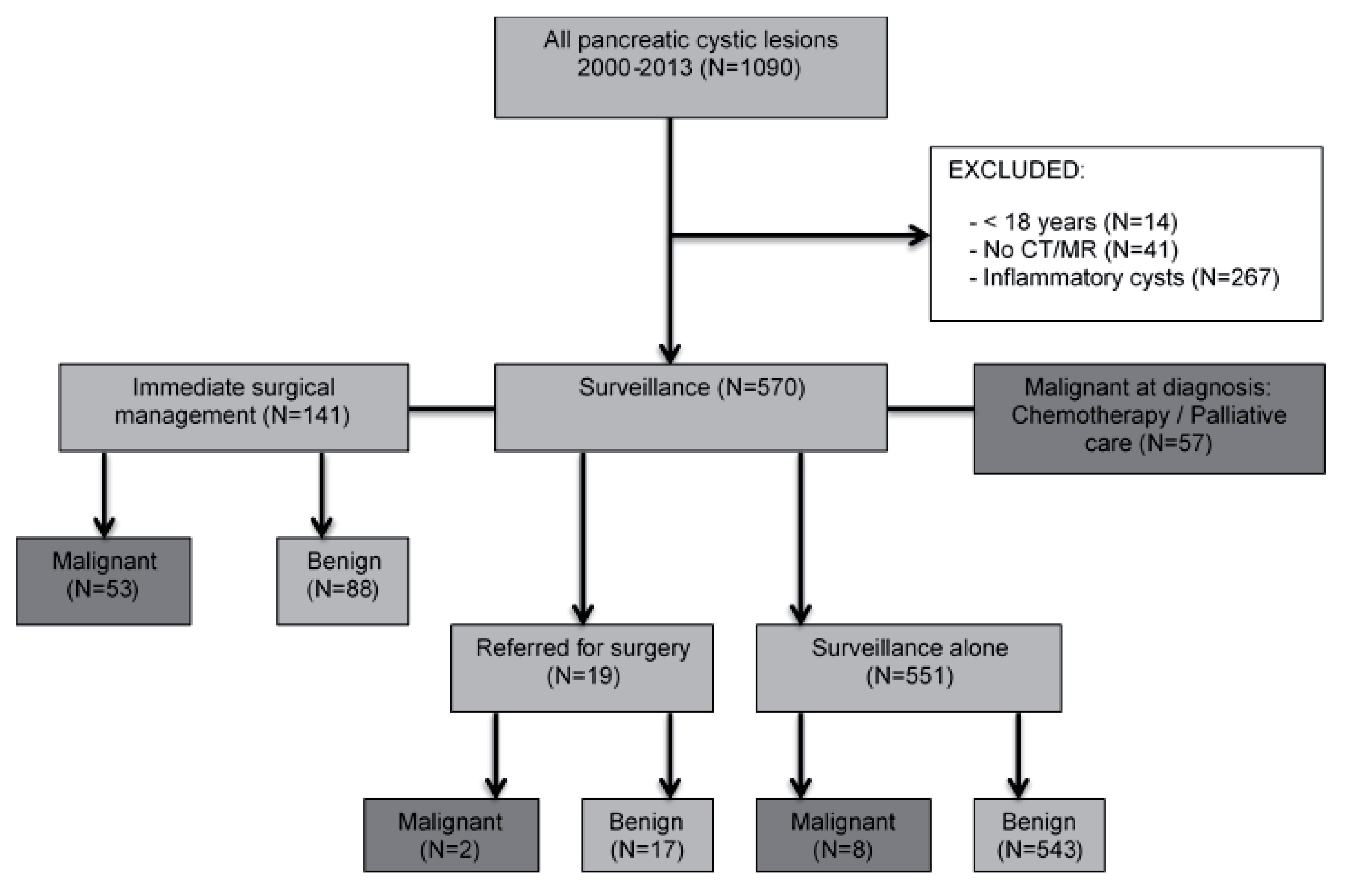
Management of pancreatic cystic lesions (PCL) discussed at multidisciplinary meeting (MDT) between 2000 and 2013.

During the 14-year study period, 1090 patients with PCL were evaluated at the HPB MDT with rates new referrals increasing annually. 14 patients were under 18 years and were excluded from the study, as were 41 patients who had had a PCL identified on EUS but without available cross-sectional imaging. During follow-up of >12 months, 267 cysts were confirmed as pseudocysts, necessitating endoscopic or percutaneous drainage, and were also excluded. The study therefore included 768 patients, with a PCL necessitating surgery, oncologic management or surveillance [ Figure 1].

### Diagnostic work-up prior to MDT

97% (743/768) of patients assessed at the MDT had had a CT; the remaining 3% of patients underwent an MR / MRCP. 34% (259/768) of patients had both a CT and MRI as part of their diagnostic work-up. In patients with an indeterminate PCL, or worrisome feature on cross-sectional imaging, an EUS was performed in 39% (301/768), an ERCP in 9% (67/768) and a percutaneous biopsy in 4% (34/768).

### Surgery

Of the 768 patient included in the study, 141 (18%) were referred for immediate surgical resection; a further 19 who were initially managed by surveillance eventually underwent pancreatic resection. 79 patients had an open or laparoscopic distal pancreatectomy with or without splenectomy, 65 had a Whipple’s or pylorus-preserving pancreaticoduodenectomy, 10 had a total pancreatectomy and the remaining 6 patients had an enucleation [ Table 1a and Table 1b]. The 30-day mortality following pancreatic resection for a PCL was 1% (2/160). Post-operatively, patients were followed up for a median of 15 (range 0–121) months.

Of the 56 patients who underwent pancreatic resection for malignant disease, 16 received adjuvant chemotherapy and 20% (11/56) died during follow-up. Of these, 9 cases were as a result of pancreatic cancer, one patient died unexpectedly while in hospital from an undetermined cause and one died from metastatic breast cancer.

Median survival following resection of a malignant PCL was 8 (range: 0–19) months for PDAC (no PCL), 16 (range: 0–91) months for a malignant IPMN, 32 (range: 5–84) months for a PNET, 26 (range: 7–35) months for a SPPN and 43 (range: 11–69) months for a malignant MCN.

### Surveillance

**Figure 2.**
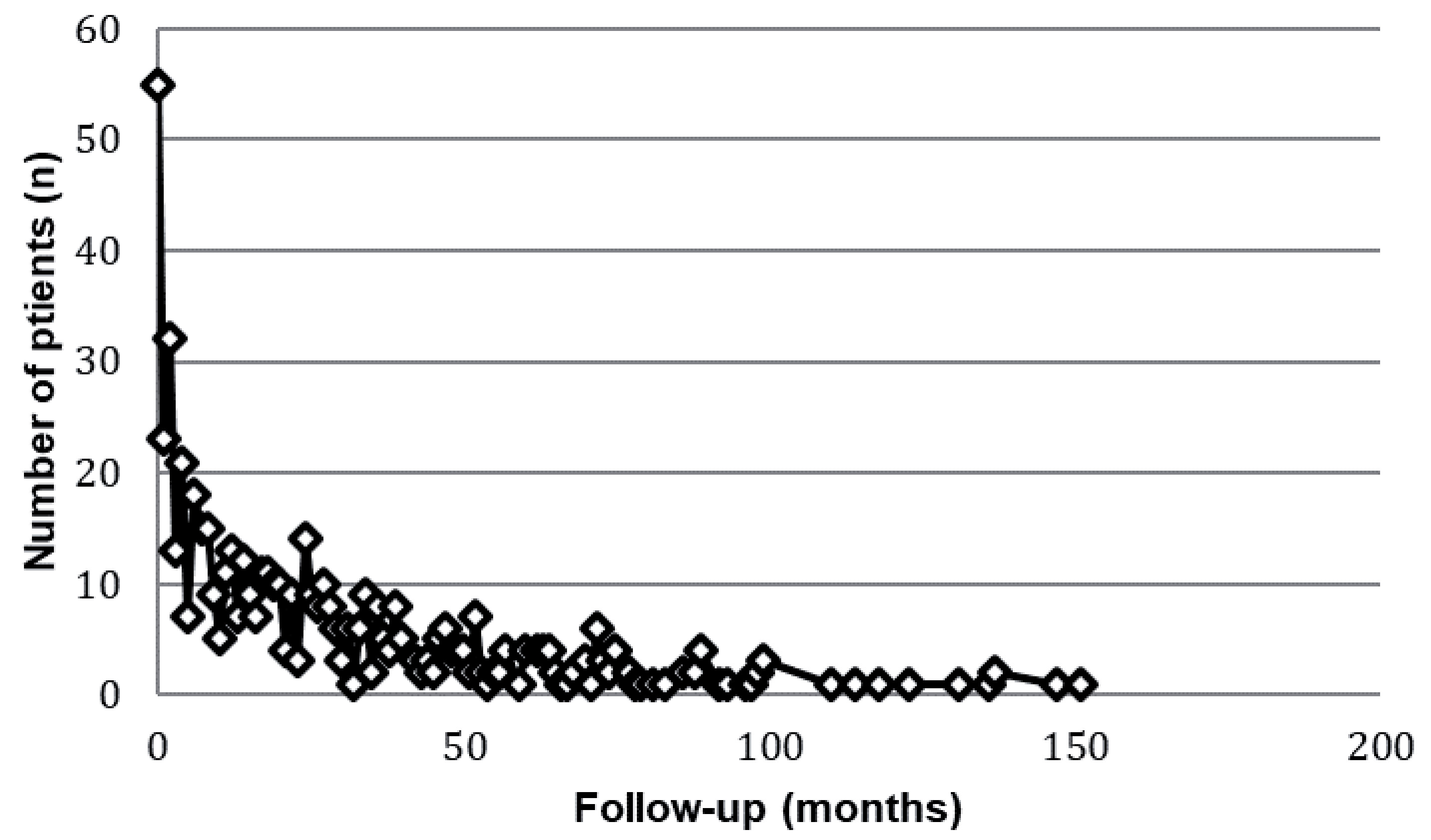
Duration of time spent in active surveillance for a pancreatic cystic lesions (PCL).

During the study period 570 patients entered the surveillance programme. The median follow-up was 85 months (range, 0–1452 months) but dropout from surveillance was considerable after 12 months [ Figure 2]. The median age of patients managed by surveillance was 67 years (range 20–92), which was older than those receiving surgical management. The median size of a cyst at entry to the surveillance programme was 20mm (range 3–130), which was smaller than all other management subtypes [ Table 2].

Of the 451 patients with serial imaging during surveillance, 76 cysts (17%) increased in size, 272 remained stable, 50 decreased in size and 54 resolved [ Table 3]. During follow up, 3% (19/570) of patients were ultimately referred to surgery and 2% (10/570) developed pancreatic cancer [ Figure 1].

Of the 10 that underwent malignant transformation, nine of the PCL increased in size in addition to all developing worrying features [ Table 4]. Seven of the 10 patients had an EUS; which was non-diagnostic in two cases and suggested benign pathology in the remaining cases. Only two of the 10 patients were ultimately referred for surgical resection; both had R0 resections and one developed recurrence at 13 months. The other eight patients were managed non-operatively, five having been discharged from active surveillance, as they were no longer fit for surgical resection. Two further patients were discharged from surveillance because the PCL was presumed to be an inflammatory cyst and one patient ultimately refused surgical intervention after developing unresectable pancreatic cancer [ Table 4].

Of the 3% of patients in surveillance who were ultimately referred for surgery, 47% (9/19) were found to have a non-mucinous, non-malignant cyst on final pathology [ Table 1b]. These patients had been in a surveillance programme for a median of 37 months prior to surgery (range: 7–64 months).

### Features of malignant transformation

**Figure 3.**
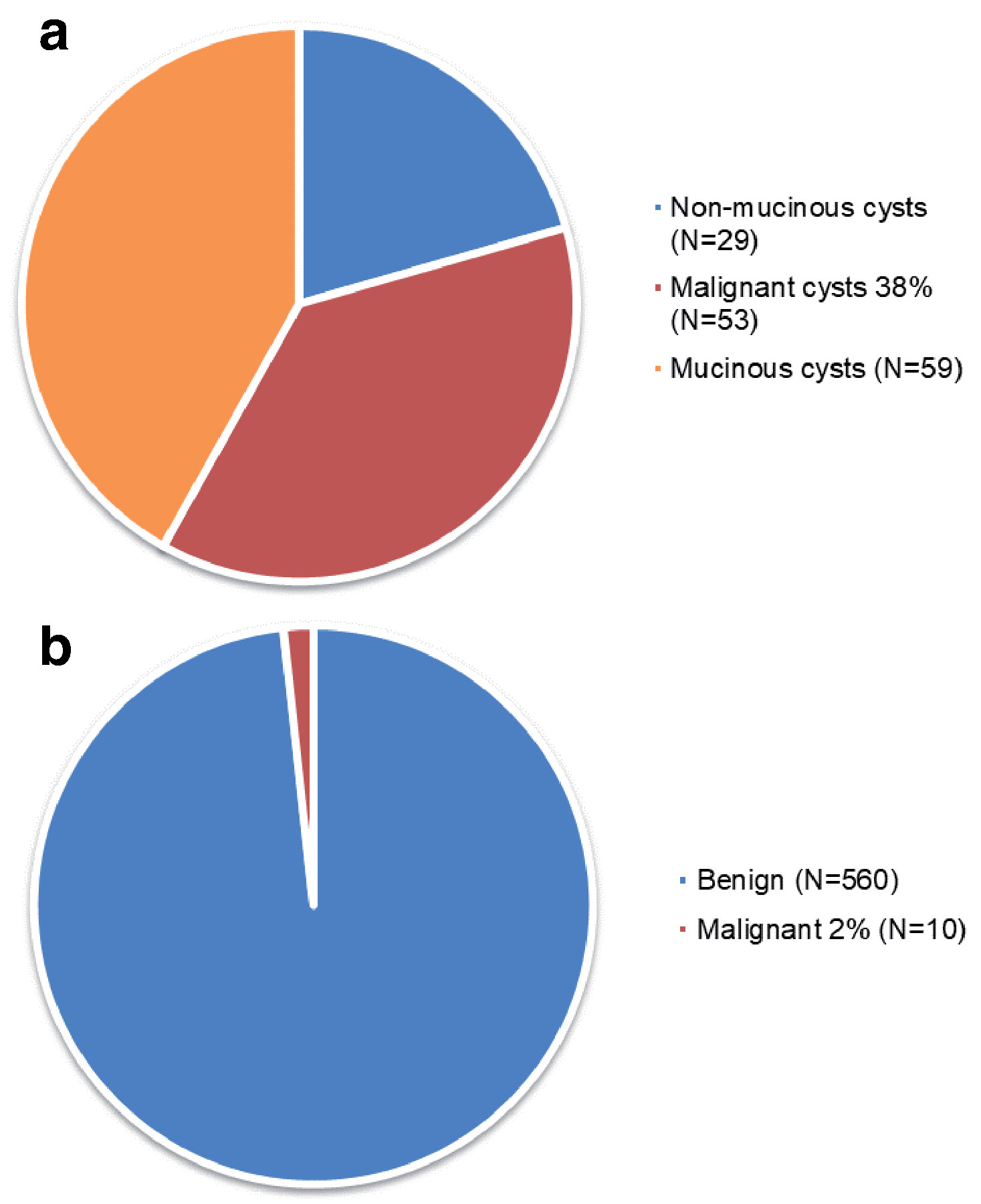
Incidence of pancreatic malignancy in surgical and surveillance cohorts (**a**) Immediate surgical management, (**b**) Surveillance: proportion that underwent malignant transformation.

**Figure 4.**
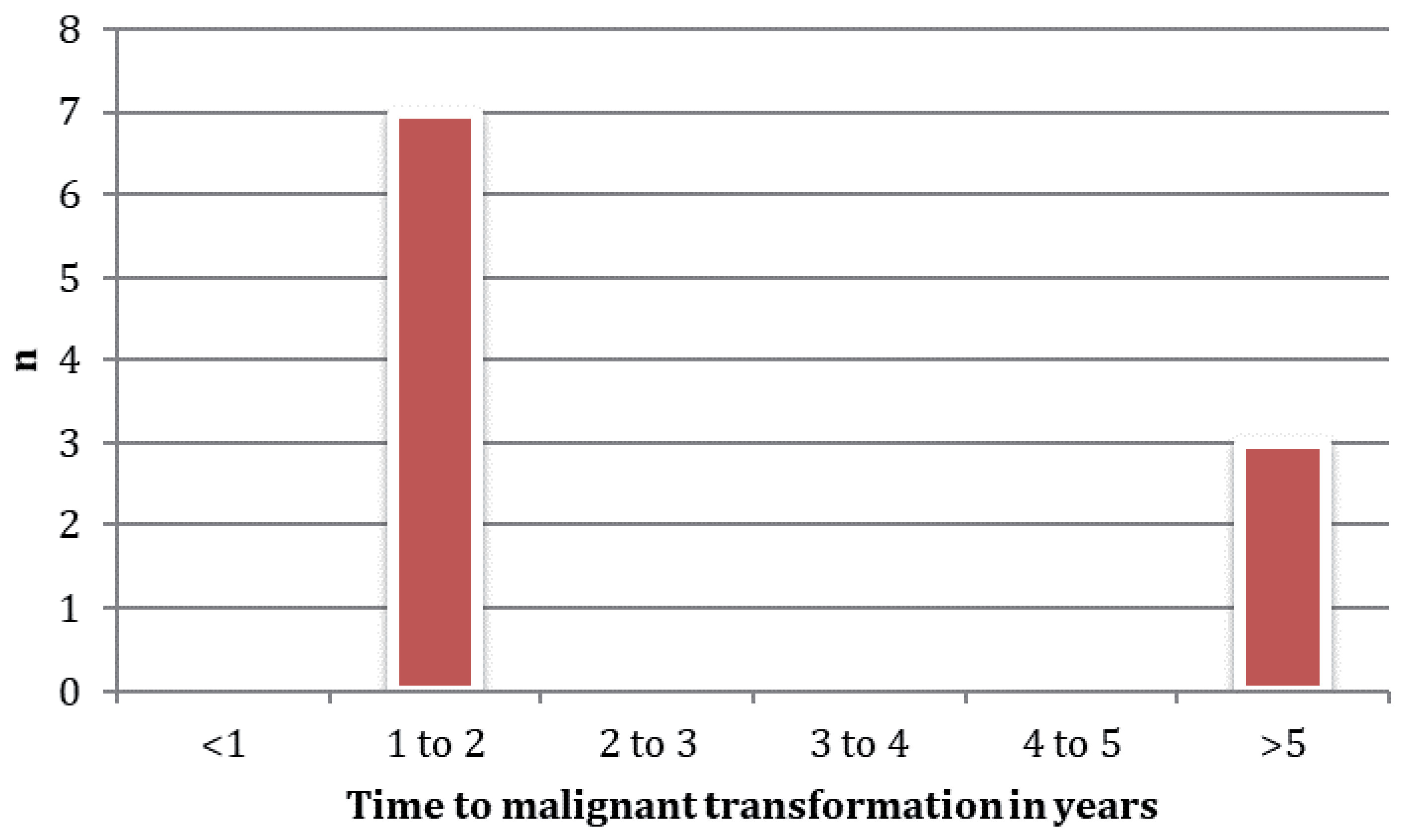
Time to malignant transformation in patients under surveillance for a pancreatic cystic lesions (PCL).

During the study period, 16% (120/768) of patients were diagnosed with pancreatic cancer of whom 46% (55/120) underwent surgical resection. Of the patients initially referred for surgery, 38% (53/141) were diagnosed with a malignant pancreatic cyst compared to 2% (10/570) in the surveillance group [ Figure 3]. 92% (110/120) of all patients with malignancy were diagnosed at the time the PCL was detected. The median age at diagnosis for a malignant PCL was 67 (23–95) years. 64% (67/105) were symptomatic. The median size of a malignant PCL at diagnosis was 35 (6–250) mm. 39% (47/120) had an associated solid component and 38% (45/120) had pancreatic duct dilation. Most patients developing malignancy did so within 2 year of diagnosis, but 30% underwent malignant transformation after more than 5 years follow up [ Figure 4].

The overall sensitivity of current diagnostic tests leading to immediate surgery for high-risk PCL (malignant or mucinous) was high (92%) but specificity was low (5%). Table 2a. and Table 2b, compares cross-sectional imaging features by management and cyst subtype. Cysts that were malignant at diagnosis or were referred for immediate surgical resection were larger than cysts managed by follow-up surveillance. A mural nodule was an exceptionally rare radiological finding in patients in this study, but a solid component was present in 42% of patients with malignant cysts managed by chemotherapy and palliative care compared to 22% of PCL referred to surgery and only 10% of PCL entering surveillance. Pancreatic and common bile duct dilatation along with lymph node enlargement were also common features of malignant cysts managed non-operatively.

**Figure 5.**
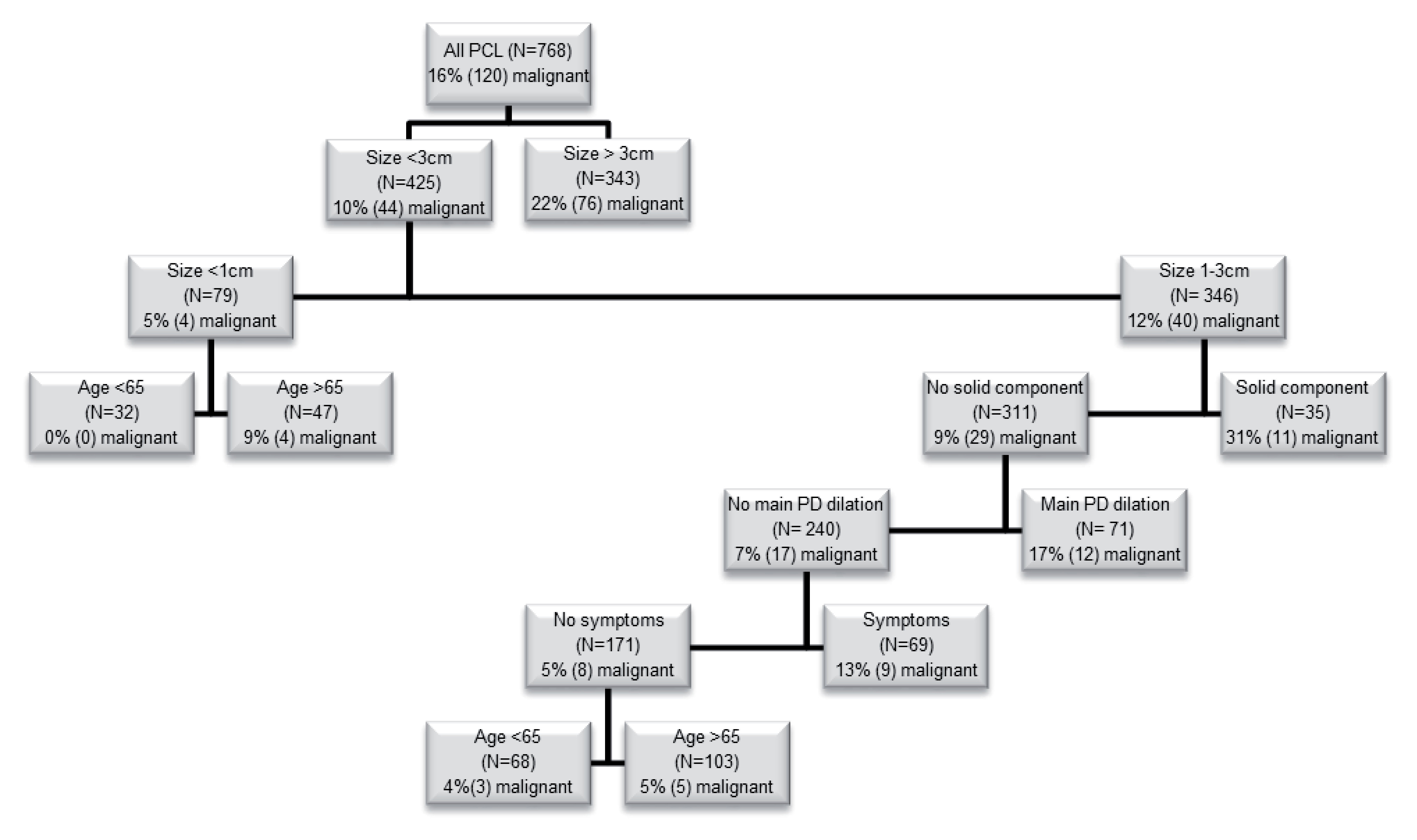
Recursive partitioning analysis: risk stratification for a PCL based on clinical and imaging features. PCL: pancreatic cystic lesion, PD: pancreatic duct.

Clinical and radiological features which suggested malignant transformation were used to formulate a decision tree model, to guide management and counsel patients with a PCL from diagnosis and though surveillance [ Figure 5].

## Discussion

In this large cohort study of patients with a PCL referred to a tertiary referral HPB centre, most malignant lesions were detected within 1–2 years of diagnosis with a PCL or developed after substantial follow up (>5 years). This has been reported similarly by other groups and supports long-term surveillance for patients with mucinous PCL who are fit for surgical resection^18^. As expected, patients with high risk and worrisome features who were referred for immediate surgery had much higher rates of associated malignancy than those managed by surveillance with interval imaging (38% vs. 2%). However, although pre-operative investigations had a high sensitivity for detecting malignancy, they were associated with a poor specificity and a substantial proportion of patients underwent unnecessary surgery (21% of immediate and 47% of delayed pancreatic resections had completely benign disease e.g. SCN which would have never undergone malignant transformation). Other groups have reported similar findings with pre-operative cross-sectional imaging correlating with surgical pathological findings in only 30–74% of cases^19^. This is significant in this population as pancreatic resection has an associated morbidity (20.8–59%) and mortality of 0–7.1% (1% in our cohort) in high volume centres^20^. These findings suggests that current diagnostic tools used in pre-operative workup are imperfect^21^,^22^, and improved tests and novel diagnostic adjuncts are required.

Pre-operative evaluation of PCL in this study was primarily dependent on clinical and radiological features, which were assessed during a weekly MDT meeting. Some groups have found serum CA 19-9 to be helpful in defining malignant transformation in PCL^23^, but in this cohort it was not consistently elevated in any of the malignant subtypes. Cross-sectional imaging (CT and MR/MRCP) of the pancreas can effectively visualise septations, calcification, pancreatic or biliary duct dilation and the presence of solid components or enhancing mural nodules. In this cohort as in other studies^14^, size alone was found to be a poor predictor of malignant transformation; the median size of resected benign IPMNs was larger than malignant IPMNs and with the exception of MCNs, the median size of all malignant PCL that were resected was less than 3cm. In this cohort, other features which suggested malignant transformation included the presence of associated symptoms, older age, solid component, pancreatic duct dilation and increasing size.

Given that cross-sectional imaging is an imperfect diagnostic tool it is often complemented by EUS in many centres, in order to provide additional imaging information and cytological and/or biochemical analysis of the cyst fluid. However there are also limitations to this technique and in this study no cases of malignant transformation were diagnosed cytologically pre-operatively. Low cytological yields from PCL have also been reported by a number of other groups^22^,^24^–^28^. Recently novel adjuncts to the EUS technique such as confocal endomicroscopy and through the needle biopsy forceps have been developed to improve the diagnostic accuracy of the technique^29^,^30^. Other groups have found that genetic markers in cyst fluid can aid differentiation of mucinous lesions^31^. These techniques may be incorporated in to diagnostic algorithms for PCL in the future.

Of the 10 patients in our surveillance group who ultimately developed pancreatic cancer, five had been discharged from active surveillance as they were no longer fit for surgical resection and two because they were thought to have inflammatory lesions. Of the two referred for surgery, one underwent a curative Whipple’s resection and the other had a R0 total pancreatectomy and splenectomy but developed recurrence 13 months later. Patients undergoing malignant transformation were older than the majority of patients in surveillance so a lead-time bias may account for the initial peak in malignant transformation seen in this study. Large international surveillance cohorts have found similar rates of malignant transformation to that seen in this cohort^11^,^29^–^31^.

## Strengths and limitations

This study has several strengths; describing the clinical and radiological characteristics of a large cohort of patients with PCL, including a substantial surveillance cohort, with longterm follow up. An individual chart review of all patient electronic records facilitated accurate cyst diagnosis and characterisation in each case. This study unlike other cohorts^18^, included patients with a history of acute and/or chronic pancreatitis. 33% of patients with a benign IPMN and 25% of patients with a malignant IPMN had a history of acute or chronic pancreatitis, confirming it is not a feature of inflammatory PCLs alone.

However, there were some limitations associated with this study. The overall rate of malignancy in this study was 16% (120/768). Other large HPB centres have reported similar rates of malignancy^11^,^30^,^31^ and probably reflects increased rates of high risk referrals from surrounding hospitals as rates are considerably higher than those reported by community based population cohorts, suggesting that there is a cohort of largely low-risk patients who are managed outside of HPB centres^18^. This is important when interpreting the results of this study and the applicability of the recursive partitioning model.

In the surveillance cohort most patients did not undergo EUS or surgical resection as part of their management, therefore it was impossible to reliably classify cysts by histologic subtype so low-grade malignancy may have been under diagnosed. Although the study was conducted over a 14-year period, median follow-up in the surveillance cohort was 18 months, which may not have been long enough to capture all cases of malignant transformation and further longitudinal studies are needed to assess the long-term risk of cyst-related malignancy in this population.

## Conclusions

In this large surveillance cohort from a tertiary referral HPB centre the overall rate of malignancy in PCL was 16%, which is lower than most surgical series but higher than community based studies. The majority of malignant lesions (92%) were detected at the time of diagnosis. The sensitivity of current diagnostic tests leading to immediate surgery for high-risk PCL (malignant or mucinous) was high (92%) but specificity was low (just 5%). Surveillance of PCL without high-risk features was associated with a low incidence of cancer development (2%) supporting the use of worrisome clinical and radiological features (older age, symptoms, increasing size of the lesion and the presence of a solid component) in the initial stratification of PCL.

## Data availability

Raw data used in this study can be found at Open Science Framework repository.

OSF: Dataset 1. UCL Pancreatic Cyst Registry, https://doi.org/10.17605/OSF.IO/7UPDM^32^

License: CC0 1.0 Universal

## Figures and Tables

**Table 1a.** Surgical resections performed.

Surgery (N=160)	N
Distal pancreatectomy +/- splenectomy	78
Pylorus-Preserving Pancreaticoduodenectomy (Whipple’s Procedure)	65
Total pancreatectomy +/- splenectomy	11
Local excision	3
Distal pancreatectomy and splenectomy with subsequent completion pancreatectomy	3

**Table 1b.** Pathological diagnosis of patients undergoing immediate and delayed surgical management of a pancreatic cystic lesions (PCL).

Immediate surgical management (N=141)			
BENIGN – 62% (88/141)	N	MALIGNANT 38% (53/141)	N
IPMN (low-high grade dysplasia)	39	Malignant IPMN	16
Serous cystadenoma	19	Pancreatic neuroendocrine tumour	12
Mucinous cystic neoplasm (low-high grade dysplasia)	16	PDAC – cystic degeneration	8
Pseudocyst	9	Solid pseuopapillary neoplasm	7
Indeterminate cystadenoma	1	Mucinous cystic adenocarcinoma	5
Benign cystic teratoma (dermoid)	1	MCN adjacent to PDAC	1
Benign vascular lesion	1	Pancreatic metastasis (renal)	1
Mucinous non-neoplastic cyst	1	Pancreatic tumour with features of PDAC and PNET	1
Mild chronic pancreatitis + ductal dilation + mucinous concreations	1	Gastrointestinal stromal tumour	1
		Pancreatic desmoid tumour	1
Delayed surgical management (N=19)			
BENIGN – 89% (17/19)	N	MALIGNANT – 11% (2/19)	N
IPMN (low-high grade dysplasia)	5	Malignant IPMN	1
Serous cystadenoma	5	PDAC – cystic degeneration	1
Mucinous cystic neoplasm (low-high grade dysplasia)	3		
Pseudocyst	2		
Lymphoepithelial cyst	2		

IPMN: Intraductal papillary mucinous neoplasm, PDAC: pancreatic ductal adenocarcinoma

**Table 2a.** Comparison of clinical features by management subtype and cyst subtype for resected lesions.

Cyst	N	Median Age (range)	Male	Female	Symptoms	Clinical history of pancreatitis	Previous cancer	Family history of PDAC / syndrome	Median CA 19–9	Range CA 19–9
MANAGEMENT										
**Immediate surgical** **management**	141	61 (23–83)	40% (56)	60% (85)	50% (64)	23% (31)	11% (15)	4% (6)	15.3	(1.4–5604)
**Surveillance**	570	67 (20–92)	47% (266)	53% (304)	36% (182)	27% (153)	25% (142)	5% (30)	11.4	(<1–2102)
**Chemotherapy / Palliative care** **(malignant at presentation)**	57	69 (43–95)	63% (36)	37% (21)	76% (40)	20% (11)	16% (9)	4% (2)	106	(<1–4981)
SURGERY – BENIGN										
**IPMN (benign)**	44	65 (42–82)	52% (23)	48% (21)	49% (18)	33% (13)	12% (5)	0% (0)	15.75	(<1–460)
**MCN**	20	60 (27–76)	5% (1)	95% (19)	50% (7)	16% (3)	5% (1)	0% (0)	15	(1.4–36)
**SCA**	24	68 (49–78)	12% (3)	88% (21)	18% (3)	0% (0)	17% (4)	4% (1)	6.5	(1.4–49)
**Pseudocyst**	11	50 (34–66)	55% (6)	45% (5)	70% (7)	73% (8)	0% (0)	9% (1)	32	(8.8–5604)
**SPN**	7	28 (23–49)	0% (0)	100% (7)	33% (2)	14% (1)	0% (0)	0% (0)	6.45	(3.5–17.4)
SURGERY – MALIGNANT										
**IPMN (malignant)**	17	72 (54–81)	47% (8)	53% (9)	79% (11)	25% (4)	19% (3)	13% (2)	10.8	(8–19.6)
**PNET**	12	55 (36–77)	50% (6)	50% (6)	17% (2)	0% (0)	17% (2)	8% (1)	32.3	(16.9–108.2)
**PDAC**	9	68 (54–77)	55% (5)	44% (4)	75% (6)	33% (3)	0% (0)	0% (0)	37	(6–119.6)
**MCN (malignant)**	5	53 (41–69)	40% (2)	60% (3)	75% (3)	20% (1)	0% (0)	0% (0)	6	6

IPMN: Intraductal papillary mucinous neoplasm, PDAC: pancreatic ductal adenocarcinoma, MCN: Mucinous cystic neoplasm, SCA: Serous cystadenoma, SPN: Solid pseudopapillary neoplasm, PNET: Pancreatic neuroendocrine tumour

**Table 2b.** Comparison of cross-sectional imaging features by management subtype and cyst subtype in resected pancreatic cystic lesions (PCL).

Cyst	N	Median Size + range (mm)	Head / Neck	Body / Tail	Multiple pancreatic cysts	Solid component	Septations	Acute pancreatitis	Chronic pancreatitis	PD dilatation (>5mm)	PD com	CBD dilatation	LN enlargement	Vascular compromise	Concomitant cysts in other organs
MANAGEMENT															
**Immediate** **surgical** **management**	141	33 (3-230)	41% (58)	59% (83)	14% (20)	22% (31)	9% (13)	4% (5)	18% (25)	28% (39)	8% (11)	14% (19)	6% (9)	4% (6)	27% (38)
**Surveillance**	570	20 (3-130)	56% (315)	44% (245)	35% (197)	10% (59)	11% (65)	11% (64)	27% (154)	27% (151)	10% (56)	13% (72)	6% (35)	3% (14)	35% (202)
**Chemotherapy/** **Palliative care** **(malignant at** **presentation)**	57	41 (7-250)	70% (40)	30% (17)	23% (13)	42% (24)	7% (4)	9% (5)	25% (14)	40% (23)	2% (1)	39% (22)	12% (7)	9% (5)	21% (12)
SURGERY - BENIGN															
**IPMN (benign)**	44	30 (11-130)	61% (27)	39% (17)	18% (8)	11% (5)	11% (5)	2% (1)	21% (9)	43% (19)	25% (11)	11% (5)	7% (3)	2% (1)	27% (12)
**MCN**	20	42.5 (18-120)	20% (4)	80% (16)	0% (0)	35% (7)	30% (6)	0% (0)	10% (2)	10% (2)	10% (2)	0% (0)	0% (0)	5% (1)	25% (5)
**SCA**	24	42.5 (14-159)	29% (7)	71% (17)	8% (2)	25% (6)	13% (3)	0% (0)	17% (4)	4% (1)	4% (1)	4% (1)	0% (0)	0% (0)	25% (6)
**Pseudocyst**	11	36 (20-90)	64% (7)	36% (4)	46% (5)	9% (1)	0% (0)	55% (6)	46% (5)	36% (4)	0% (0)	9% (1)	18% (2)	27% (3)	0% (0)
**SPPN**	7	59 (20-150)	0% (0)	100% (7)	0% (0)	29% (2)	0% (0)	0% (0)	0% (0)	0% (0)	0% (0)	0% (0)	14% (1)	0% (0)	14% (1)
SURGERY - MALIGNANT															
**IPMN** **(malignant)**	17	23 (15-56)	53% (9)	47% (8)	29% (5)	18% (3)	0% (0)	6% (1)	29% (5)	47% (8)	0% (0)	47% (8)	0% (0)	0% (0)	53% (9)
**PNET**	12	23.5 (15-94)	25% (3)	75% (9)	17% (2)	33% (4)	0% (0)	0% (0)	0% (0)	0% (0)	17% (2)	0% (0)	8% (1)	0% (0)	33% (4)
**PDAC**	9	25 (15-59)	44% (4)	55% (5)	11% (1)	44% (4)	11% (1)	0% (0)	11% (1)	67% (6)	11% (1)	44% (4)	22% (2)	0% (0)	22% (2)
**MCA**	5	120 (23-230)	40% (2)	60% (3)	0% (0)	40% (2)	0% (0)	0% (0)	40% (2)	20% (1)	0% (0/	0% (0)	0% (0)	20% (1)	0% (0)

IPMN: Intraductal papillary mucinous neoplasm, PDAC: pancreatic ductal adenocarcinoma, MCN: Mucinous cystic neoplasm, SCA: Serous cystadenoma, SPN: Solid pseudopapillary neoplasm, PNET: Pancreatic neuroendocrine tumour, PD com: PD communication

**Table 3.** Proportion of pancreatic cystic lesions (PCL), which increased, decreased, remained stable or resolved while in surveillance with interval imaging.

	N = 452	Number PDAC	Median length of follow up	Range	Referred to surgery	Currently in active follow up
**Increased**	76	9	29	(0–137)	13 (1 malignant)	21
**Stable**	272	1	22	(0–151)	5 (1 malignant)	69
**Decreased**	50	0	24	(2–83)	1	12
**Resolved**	54	0	26	(3–147)	0	2

PDAC: Pancreatic ductal adenocarcinoma

**Table 4.** Characteristics of patients who developed malignant transformation during surveillance.

Surgical management					
	Age	Sex	Time to malignant transformation from diagnosis (months)	Route to diagnosis	Management
**1**	77	M	18	Investigations for recurrent pancreatitis revealed a 2cm cyst in the uncinate. Entered surveillance, CA 19-9 rising 69.9 IU/ml. EUS-FNA revealed the cyst was communicating with a dilated main PD. Cytology non-diagnostic. ERCP– pathognomonic findings of MD-IPMN.	**Surgery:** Whipples. **Histology:** T2N0MXR0 tumour arising from a MD-IPMN. **Outcome:** No recurrence during 20-months of follow-up.
**2**	68	F	18	Imaging following acute necrotising gallstone pancreatitis revealed a 5.9cm cyst in the head of the pancreas with dilated main PD. Thought to be a symptomatic pseudocyst so a EUS guided cystenterostomy was performed. Following removal of the stents a small cyst persisted which had a solid component. CA 19–9 rising (1869.0 IU/ml). Repeat EUS-FNA: cytology consistent with a pseudocyst but cyst fluid CEA 105 ng/ml, amylase 1598 IU/L.	**Surgery:** Total pancreatectomy + splenectomy + PV reconstruction **Histology:** T3N1 (1/24) MxR0 PDAC + pseudocyst. **Outcome:** Adjuvant chemotherapy with gemcitabine. 13 months on PET-CT confirms recurrent disease – no further chemotherapy, asymptomatic.
Non-operative management					
	Age	Sex	Time to malignant transformation from diagnosis (months)	Route to diagnosis	Management
**1**	75	M	18	Right hemicolectomy for a Dukes B colorectal cancer, complicated by an anastomotic leak and prolonged ITU stay. Follow-up imaging revealed an incidental 23mm cyst in the pancreatic tail. EUS-FNA – cytology: atypical cells consistent with IPMN. 14 months later presented with jaundice. Cyst had increased to 3cm + solid component and dilated main PD. CA 19-9 rising (1879.0 IU/ml). Further EUS-FNA; cytology – atypia, histology - IPMN.	Resectable disease but patient refused pancreatic surgery. ERCP + metal stent inserted. Patient died 4 months later.
**2**	78	F	24	Admitted with deranged LFTs and abdominal pain. Imaging revealed cirrhosis and chronic pancreatitis + 12cm PCL with septations and a solid component. Developed nausea and weight loss so underwent percutaneous drainage of a presumed pseudocyst cyst at a local hospital. Follow-up imaging revealed unresectable PDAC with vascular incasement. EUS-FNA – cytology: well- differentiated PNET but IHC not supportive, CEA 36223 ug/L. Amylase < 3 IU/L.	Unresectable disease. Palliative care – died 3 months later.
**3**	71	F	76	Imaging for autoimmune hepatitis revealed multiple incidental PCL with features of chronic pancreatitis. Thought to be multiple pseudocysts and therefore not actively followed-up. Patient requested a second opinion and when reimaged lesions had undergone malignant transformation.	Multiple comorbidities unfit for surgical resection – tissue diagnosis not pursued. Palliative care – subsequently died.
**4**	70	M	62	Family history of PDAC. Abdominal imaging for renal calculi revealed a 35mm cyst is the head of the pancreas with a dilated main PD and multiple other cysts. EUS-FNA; cytology consistent with low grade IPMN. CA 19-9 66 IU/ml. Discharged from active surveillance because of comorbidity after 18 months. Recommenced after 23 months & had developed a metastatic liver lesion of upper GI origin.	Unresectable disease. Palliative cisplatin + gemcitabine chemotherapy. Died 36 months later
**5**	81	M	24	Investigated for deteriorating blood sugars (recently diagnosed Type 2 DM). Abdominal CT: dilated PD without cause. EUS-FNA: 8mm multiloculated cyst in the pancreatic tail, with mural nodule. Cytology: possible mucin-secreting tumour but non-diagnostic. CA 19-9 101 IU/ml. Discharged from active surveillance as no longer a fit for surgical resection. Represented with metastatic PDAC 6 months later. ERCP + biliary brushings: IPMN with atypia.	Unresectable disease. Palliative care – subsequently died 36 months later.
**6**	71	F	19	Background of pancreatic trauma in 1971, requiring pancreatic surgery + drainage. Investigated for faecal inconsonance, colonic polyps and exocrine insufficiency with a CT pneumocolon. Found to a dilated main PD + 14mm cyst in the pancreatic tail, presumed due to trauma. Intermittent surveillance with colonic polyp surveillance via CT over 19 months. Developed significant weight loss and repeat imaging revealed unresectable disease. Cytology from pleural aspirate confirmed metastatic adenocarcinoma (? PNET).	Unresectable disease, palliative care, died 8 months after diagnosis.
**7**	78	F	73	Right hemicolectomy for Dukes B tumour, T3N0M0. During follow-up noted to have a dilated main PD. Over time became associated with a cystic and then a solid lesion. CA 19-9 rising (526.2 IU/ml). August 2012 – cytology from EUS- FNA suggestive of chronic pancreatitis but percutaneous biopsy confirmed moderately differentiated PDAC.	Locally advanced disease but unfit for surgical resection because of comorbidities. No chemotherapy, clinically stable 26 months after diagnosis.
**8**	87	M	12	CT pneumocolon for abdominal pain and diarrhoea revealed a dilated main PD and side branches with retroperitoneal LNs. Stable on imaging for 11 months then represented with jaundice and cholangitis. CA19-9 2102 IU/ml. ERCP + brushings – non diagnostic.	Unresectable disease. Multiple comorbidities. Refused chemotherapy. Histological diagnosis not pursued. Palliative care – subsequently died.

PDAC: Pancreatic ductal adenocarcinoma. PD: Pancreatic duct. EUS-FNA: Endoscopic ultrasound and fine needle aspiration. ERCP: Endoscopic retrograde cholangiopancreatography. LN: Lymph nodes. PCL: Pancreatic cystic lesion, DM: Diabetes mellitus, IHC: immunohistochemistry
