# Supplementary material for: Clinical and radiological features that predict malignant transformation in cystic lesions of the pancreas: a retrospective case note review
Source: AMRC Open Res. Author manuscript; Available in PMC 2020 Apr 22. (PMC7176489; doi:10.12688/amrcopenres.12860.2)
Supplement: Peer review history

# Peer review: Reviewer response for version 2

**DOI:** 10.21956/amrcopenres.13960.r26480

**Published:** 2020-01-20

**Authors:** Luca Barresi

**Affiliations:** 1 Endoscopy Service, Department of Diagnostic and Therapeutic Services, IRCCS-ISMETT (Mediterranean Institute for Transplantation and Highly Specialized Therapies), Palermo, Italy

The authors correctly interpreted my suggestions and modified the discussion of their study accordingly.

I confirm that I have read this submission and believe that I have an appropriate level of expertise to confirm that it is of an acceptable scientific standard.

# Peer review: Reviewer response for version 1

**DOI:** 10.21956/amrcopenres.13926.r26472

**Published:** 2019-09-09

**Authors:** Luca Barresi

**Affiliations:** 1 Endoscopy Service, Department of Diagnostic and Therapeutic Services, IRCCS-ISMETT (Mediterranean Institute for Transplantation and Highly Specialized Therapies), Palermo, Italy

PCLs are more frequently being diagnosed due to the increased use of advanced radiological and endoscopic techniques. Often these lesions are asymptomatic and discovered incidentally, and only a small part will evolve towards aggressive lesions with metastatic potential. The problem of how to manage these lesions is discussed worldwide, and the continual publication of new diagnostic algorithms and guidelines is the best evidence that an approach shared by all clinicians is not currently available. In fact, both the diagnosis for type of lesion and the risk stratification for malignant evolution are currently very different in different centers, and often linked to the expertise available locally.

 The study presented by Dr Keane et al. is a further confirmation of what has already been highlighted in many previous studies, namely that the stratification of the risk of malignancy using only the clinic and radiological features, in particular the CT scan, will inevitably lead to a high number of false positives, which will result in a considerable number of unnecessary pancreatectomies, and burdened by the known consequences of this surgery.

 The study by our English colleagues confirms, in a large cohort of patients, against a high sensitivity, the low specificity of the clinical-radiological approach. The study is certainly well written, shows clear and detailed results, and supports the conclusions drawn by the authors. The statistical data are adequate for the evaluations carried out.

 Noteworthy is that the follow-up of 18 months (on average) is too short to highlight the malignant evolution of these lesions, which have a very slow developing potential, as rightly pointed out by the authors themselves.

 In addition, the authors report in the Abstract a selection of patients with at least 5 years of follow-up, which does not seem correct since the average follow up reported is only 18 months (to be corrected).

 However, in addition to the main purpose of the study, the data presented confirm other interesting information that deserves to be emphasized:

1. It is interesting to note that in this study the majority of malignant lesions (92%) were highlighted at the first diagnosis of PCL. This was already observed in a previous study (reference in the study No. 18), where 74% of the malignant lesions were found within the first 3 months of initial diagnosis. This observation, translated into practical terms, means that the first approach to the patient with PCLs is fundamental because the clinician has the highest probability of observing the lesions that deserve surgical treatment. For this reason, the approach of our centre is to carefully study the lesions at the first diagnosis, in particular those with “worrisome features”, and to plan the first follow-up, for lesions that are not sent immediately to the surgery, at a short distance (usually 3 months, but certainly not more than 6) considering this control a sort of extension of the initial evaluation.
2. Despite point one, some lesions may degenerate even after having remained stable for more than 5 years. The study shows, in fact, a malignant evolution over 5 years from diagnosis in 30% of the lesions that degenerate during follow-up. It is also necessary to remember the possibility of occurrence of a pancreatic cancer concomitant but distinct from IPMN, an event not infrequent in this type of lesion, and also possible after the first 5 years of follow-up (3.5% at 10 years, and 12% at 15 years from the initial diagnosis)^1^. These data confirm that the guidelines of the American Gastroenterology Association (AGA)^2^, whose recommendation to suspend the follow-up after 5 years of stability of the PCLs is risky and lacks justification. Follow-up should therefore continue indefinitely, as long as the patient remains fit for surgery.
3. The size of PCLs is of relative relevance to the stratification of the risk of malignancy (threefold increase in the malignancy risk for lesions >3 cm, instead the presence of mural nodules carries an eightfold increase in the malignancy risk). This has been highlighted in several studies, which is why the most widely used guidelines in the world, the International Consensus Guideline of Sendai-Fukuoka, last revised in 2017, have downgraded the size > 3 cm from "indication to surgery" of the first draft of 2006 (Sendai Guidelines) to lesions with "worrisome features", which means lesions with indication to perform other investigations (in particular an EUS-FNA), already in the 2012 revision (Fukuoka Guidelines), thus reducing the risk of unnecessary interventions.
4. Cytology on cystic fluid has a low diagnostic yield because of the paucity of cells dispersed in the cystic fluid. This is known and confirmed by multiple studies, which is why other methods of tissue acquisition (in particular EUS-through-the-needle biopsy) or *in situ* evaluation (with confocal laser endomicroscopy) have been used, with very encouraging results.
5. Both benign and malignant IPMN can occur with a certain frequency (about 25-30%) with acute or chronic pancreatitis. Therefore, finding a cystic lesion in a patient with recent acute (or chronic) pancreatitis is not diagnostic in all cases of pseudocyst. This is relevant and implies that the diagnostic evaluation should also be conducted on these PCLs.
6. Follow-up of lesions without "high risk stigmata" is a reasonable and relatively safe choice since only a small percentage (about 2% in this study) will degenerate during follow-up, as previously reported [3].
7. The presence of multiple (and sometimes contradictory) guidelines, and the need to adapt the approach to patients with PCLs certainly makes a shared approach valid and advisable through a multidisciplinary team that will evaluate and indicate the best management for the indeterminate and most suspected of degeneration PCLs. 

In addition to these strengths of the study there are, however, also weaknesses:

1. In the study, magnetic resonance imaging (MRI) was used in only 34% of cases, with a prevalence of CT scanning. This may have reduced the diagnostic capacity of the radiologists since the spatial resolution of the MRI, and therefore the evaluation of the internal components of the PCLs, as well as the evaluation of the connection with the main pancreatic duct, is decidedly better with MRI. In addition, MRI easily highlights debris in pseudocyst, which is a specific aspect of pancreatic-peripancreatic collection (walled off necrosis), and absent in neoplastic PCLs. This aspect is not usually visible in CT scans.
2. The same goes for the limited use of the EUS in the study’s cohort (39%, and only in patients with worrisome features), which has an even greater capacity than MRI in the evaluation of the internal structure of the PCLs to highlight possible microcystic components (highly suspect for serous cystadenoma, which were 15% of patients undergoing surgery), debris, small wall nodules (with the possibility also of differential diagnosis between mucus plug and epithelial nodules by means of contrast media) and evaluation of the connection with the main pancreatic duct for the diagnosis of IPMN.

 I think it is highly likely that the use of such methods in all patients of the study would have significantly reduced the number of false positives and unnecessary surgery (in the study among the lesions that were indicated for surgery, 15% were serous cystadenomas and 7% pseudocyst).

 But what could have contributed most to a better stratification of risk are the new methods, of which practically no mention is made in the work.

 Even if needle-based endomicroscopy and EUS-through-the-needle biopsy were not used in the patients of this study (obviously, these were not available in the study period), it would be useful for the readers of this article to have at least a hint of what is currently feasible for our patients with PCLs.

 Needle based endomicroscopy and tissue acquisition from the cystic wall with the EUS-through-the-needle biopsy have sufficient literature to be taken seriously as the new most credited methods for improving the diagnosis and stratification of risk for malignancy in PCLs. I strongly advise the authors to cite such methods, even briefly, underlining the importance they have assumed and will assume in the coming years.

 Though, moreover, the analysis of the cystic fluid for the CEA is certainly not a perfect method, it is worth mentioning, perhaps by associating it with the evaluation of intracystic glucose, which seems promising and complementary to the CEA, and very easy to performe.

 Last but not least, I would mention molecular biology on cystic fluid, which could be a turning point, as highlighted in recent studies, both in the diagnosis of the type of cysts and in the stratification for the risk of malignancy.

 My suggestion is to review the discussion underlining the above points (both positive and negative) to provide readers with a more complete picture of the data highlighted by the study, and open a window on the principal changes across recent years, so as to make current the information that has emerged from this interesting retrospective assessment.

I confirm that I have read this submission and believe that I have an appropriate level of expertise to confirm that it is of an acceptable scientific standard, however I have significant reservations, as outlined above.

## References

[1] OyamaH: Long-term Risk of Malignancy in Branch Duct Intraductal Papillary Mucinous Neoplasms. *Gastroenterology*.2019; 10.1053/j.gastro.2019.08.032 31473224

[2] VegeSS: American gastroenterological association institute guideline on the diagnosis and management of asymptomatic neoplastic pancreatic cysts. *Gastroenterology*.2015;148(4) : 819-22; quize12 10.1053/j.gastro.2015.01.015 25805375

[3] CrippaS: Low progression of intraductal papillary mucinous neoplasms with worrisome features and high-risk stigmata undergoing non-operative management: a mid-term follow-up analysis. *Gut*.66(3) : 495-506 10.1136/gutjnl-2015-310162 26743012

# Peer review: Reviewer response for version 1

**DOI:** 10.21956/amrcopenres.13926.r26427

**Published:** 2019-06-19

**Authors:** Ashley R. Dennison

**Affiliations:** 1 Department of Hepatobiliary and Pancreatic Surgery, Leicester General Hospital, University of Hospitals of Leicester NHS Trust, Leicester, UK

Particularly over the last two decades the increased number of cystic lesions of the pancreas (PCL) being identified has become a workload issue and the best management remains controversial. The workload problems, particularly for incidentally discovered lesions referred into tertiary centres, has prompted much discussion at National and International meetings with particular emphasis on attempts to define the cohort that could be safely managed conservatively. This is especially important with PCLs due to the essentially binary nature of the possible approaches and the consequences of inappropriate treatment. A proportion of PCLs will undergo malignant transformation and the consequences of this being missed have significant clinical, psychological and medico-legal repercussions. Conversely surgical intervention that proves to be unnecessary is costly and potentially associated with significant, avoidable morbidity and mortality. Thus a robust, evidence based approach to the management of PCLs is required to obviate these problems and reassure clinicians in lower volume centres in respect of the algorithm they employ.  

**Is the work clearly and accurately presented and does it cite the current literature?**

 The study presented by Keane *et al *represents a significant contribution to the literature from a high volume, internationally respected pancreatic unit. The presentation is clear and particularly the use of tables and diagrams helpful. Individual data are provided for those patients whose PCLs underwent malignant change while on the surveillance program. The literature is contemporary and relevant and of note is that the number is relatively small, dates back to 2004 and only contains 4 references in the last 5 years. This demonstrates the relative paucity of data and the difficulty in gathering data from a sufficient number of patients together with a meaningful follow up period. This reflects the difficulty of identifying an appropriate cohort with a sufficient length of follow up to enable clinicians to produce clear and universally acceptable guidelines.  

**Is the study design appropriate and is the work sound?**

****

 The study is a retrospective case report series over a 14 year period, the importance of which relates to the large number of patients and the accurate follow up that has been possible for a significant length of time. The size of the series with 1,090 patients identified (with 768 available for the study following exclusion of those under 18, those with no cross sectional imaging available and 267 pseudocysts requiring drainage) and the large amount of data available allows firm conclusions to be drawn.  

 In the methods section of the abstract however it is stated that *“adults who were evaluated in a tertiary hepatopancreaticobiliary unit between January 2000 – December 2013 with a confirmed PCL and followed up for at least 5 years” * whereas the median follow up in the surveillance group was 18 months (range 0 – 151) and this should be clarified.

**Are sufficient details of methods and analysis provided to allow replication by others?**

****

 The methodology is clearly described and appropriate. Detailed patient records had been electronically recorded for a sufficient length of time to ensure that a very large cohort with a detailed follow up enabling comprehensive and accurate data to be identified and collected. In addition to the usual demographic data this included family history, tumour markers, cross sectional imaging and EUS findings. This set of data is particularly valuable due to the lack of a significant change in routine clinical practice for the investigation of PCLs since the study period allowing the conclusions to be widely applicable.

****

**If applicable, is the statistical analysis and its interpretation appropriate?**

****

 The statistical methods utilised SPSS for windows and the 2-sample t test is appropriate.

**Are the source data underlying the results available to ensure full reproducibility?**

****

 The source data are not included in the paper but would be available.

****

**Are the conclusions drawn adequately supported by the results?**

****

 The conclusions are supported by the results and the findings of this study are interesting and the conclusions important. The amount of data together with the inclusion of patients with acute and chronic pancreatitis is useful and reflects the “real world” presentation of the patients at HPB MDT meetings and provides additional reassurance to clinicians that the conclusions and advice will be applicable in everyday practice. The data are detailed but of particular importance are:

  

1. Estimation of ca19,9 was not helpful in malignant PCLs.
2. Cross sectional imaging alone can identify features associated with the development of malignancy.
3. Features that were identified to indicate malignant change were consistent with those previously described in other series.
4. The sensitivity of the traditional diagnostic methods was 92% but the specificity was only 5%. 

One further interesting feature however was the size of the resected lesions. This study demonstrated that size *per se* could not be relied upon to predict malignancy and for IPMNs the size of lesions found to be malignant following resection was smaller than benign lesions and in addition the median size of all resected PCLs found to have undergone malignant change was less than 3cm. This is important specifically for those patients presenting for the first time when traditionally it was felt that malignancy was unlikely if the lesion was less than 30 – 35mm. This point is further emphasised by the poor outcome of malignant PCLs with a median survival of 8 months (0 – 19).

**Summary:**

****

 This is a well conducted study examining a large cohort of patients with PCLs which are a steadily increasing component of HPB MDTs. The management of these patients remains unclear and there is a relative paucity of data particularly examining a sufficiently large cohort with adequate follow up.

 While the study supports the safety of surveillance of PCLs without malignant features and confirms that the incidence of malignant change is small, it also found importantly that size *per se* (as distinct from increase in size during surveillance) cannot be relied upon when predicting malignancy. This is an important finding when assessing patients at their first presentation.

I confirm that I have read this submission and believe that I have an appropriate level of expertise to confirm that it is of an acceptable scientific standard.
